# Perceptions of Risk, Work, and Lifestyle Changes on Mental Health of Healthcare Workers Amidst the COVID-19 Pandemic

**DOI:** 10.3390/ijerph19095420

**Published:** 2022-04-29

**Authors:** Awatef Ergai, LeeAnna Spiva, Lin Li, Ryan Breshears, Ginny Zhan

**Affiliations:** 1 Department of Industrial and Systems Engineering, Southern Polytechnic College of Engineering and Engineering Technology, Kennesaw State University, Kennesaw, GA 30144, USA; lli19@kennesaw.edu; 2 Nursing Practice and Operations, Wellstar Health System, Atlanta, GA 30339, USA; leeanna.spiva@wellstar.org; 3 Wellstar Psychological Services, Marietta, GA 30060, USA; ryan.breshears@wellstar.org; 4 Department of Psychological Science, Norman J. Radow College of Humanities and Social Sciences, Kennesaw, GA 30144, USA; gzhan@kennesaw.edu

**Keywords:** COVID-19, mental health, anxiety, depression, distress, healthcare workers, psychological resources/services, first COVID-19 peak

## Abstract

The COVID-19 outbreak is significantly affecting the mental health of healthcare workers worldwide. This study aims to investigate the mental health outcomes of healthcare workers in a health system located in southeastern US during the first peak of the pandemic and examine the association of specific factors on the mental well-being of healthcare workers. A cross-sectional survey of 388 healthcare workers was conducted. Data were collected using a 79-item questionnaire, which included the Patient Health Questionnaire (PHQ-9) instrument, the 7-item Generalized Anxiety Disorder (GAD-7) instrument, and the 22-item Impact of Event Scale-Revised (IES-R), to assess symptoms of depression, anxiety, and general distress, respectively. Data were analyzed using descriptive, bivariate, and multivariate statistics. Accordingly, 30.1%, 28.7%, and 39.4% of respondents reported depression, anxiety, and distress symptoms, respectively. Younger workers and females reported higher mental symptomologies. We identified significant, nontraditional factors associated with depression and anxiety symptoms among healthcare workers: healthcare procedure change, concern of exposing family to COVID-19, number of missed shifts, and access to psychological resources/services. These findings emphasize the importance of providing the proper training to reduce concerns of exposing family members and psychological interventions to promote mental health well-being for healthcare workers during the stressful COVID-19 pandemic.

## 1. Introduction

In December 2019, the novel coronavirus disease known as COVID-19 was first reported in Wuhan, China [1,2]. The disease then exponentially spread throughout China and the rest of the world, becoming a global pandemic [3]. Prior research demonstrated that the most recent infectious diseases, including the severe acute respiratory syndrome (SARS), the Middle East respiratory syndrome (MERS), and the Ebola virus, caused significant mental health concerns among healthcare workers (HCWs) [4,5,6,7]. However, in the case of COVID-19, the number of infected cases and deaths has been exponentially higher than that of severe acute respiratory syndrome (SARS) [8] and Middle East respiratory syndrome (MERS) [9]. 

Accordingly, in the US and most of the world, healthcare systems face incredible challenges due to the COVID-19 pandemic. HCWs are at the front line of the COVID-19 outbreak response and, as such, are not only exposed to hazards that put them at risk of infection but to other dynamics that may affect their mental health, such as the constant rise of infected cases and deaths, shortages of personal protective equipment (PPE), increased workload, and lack of support from management [10,11]. These dynamics may contribute to their mental burden and, regardless, have dramatically affected the way people work, challenging employees’ health, well-being, and work engagement [1]. 

According to the WHO [12], the physical and mental health of HCWs is critical to the community’s successful survival during a pandemic. Therefore, there is an urgent need to assess healthcare employees’ mental health and their experiences during COVID-19, which can provide valuable insights into how to manage the current situation, plan for the recovery period, and anticipate future challenges. 

Thus, since the beginning of the pandemic, research on the prevalence of mental health disorders, such as depression, anxiety, distress, and burnout, among HCWs emerged as an important research topic worldwide with most studies focusing on medical personnel, specifically nurses’ and doctors’ experiences [5,10,13,14,15,16,17,18,19]. For example, in China, the prevalence of depression and anxiety among nurses varied from 9.4% and 8.1% [15] to 50% and 44.6% [16], respectively. In Italy, the reported prevalence of depression, anxiety, and distress among nurses was 19.8%, 8.2%, and 24.7% [17]. In the US, Serrano [18] found the prevalence of depression and anxiety among nurses during the first COVID-19 peak (30 April 2020–22 May 2020) was 19% and 31.6%, respectively. Parasad et al. [19] conducted a US cross-sectional study involving 20,947 HCWs between 28 May 2020 and 1 October 2020 and found 38% of HCWs reported anxiety/depression symptoms, and 49% suffered burnout.

Additionally, numerous studies examined the association between mental health disorders and factors such as demographic, work environment, and social characteristics [5,10,11,13,14,16,17,18,19]. Gender and age were significant demographic factors associated with both depression and anxiety [11,16,17,20,21]. Young female workers reported higher anxiety and depression symptoms [11,16,17]. Frontline HCWs engaged in direct contact with diagnosis, treatment, and/or care of COVID-19 patients were significantly associated with elevated mental health disorders [16,17,20]. Poor social support and self-efficacy were also associated with increased anxiety, depressive symptoms, and insomnia [22]. Fear of becoming infected with COVID-19 [19] and infecting family members [11,21] were also associated with elevated depression and anxiety symptoms.

However, additional factors that may be associated with mental health disorders have rarely been examined in previous studies: leadership role, communication frequency of supervisors with their constituents, number of missed shifts, access to psychological services, changes in how HCWs work due to COVID-19, procedures implemented by the health system, and lifestyle. Hence, the current study aimed to:

1. Determine the prevalence of depression, anxiety, and distress among HCWs; and
2. Examine the association of mental health disorders, namely depression, anxiety, and distress, with factors involving: (1) demographics; (2) work environment; (3) COVID-19 concerns; (4) work and procedural changes implemented by the health system; (5) access to psychological services; and (6) lifestyle changes outside of work, during the first COVID-19 peak, which corresponded with the highest healthcare system utilization level.

## 2. Materials and Methods

### 2.1. Participants

Participants included a convenience sample of HCWs employed in any of the eleven hospitals in the integrated healthcare system located in a southeastern state. Using the Roasoft sample size calculator, a sample size of at least 377 participants was required to realize a margin of error of 0.05 and a 95% confidence level [23]. A total of 441 participants accessed the survey and of those, 388 were the final number after removing those who did not consent and/or did not complete any mental health measurements. The response rates could not be quantified due to the self-selected nature of the sample. The participants’ age ranged from 20 to 60+ years with an average of 45 years. A majority of the participants were women (89.69%). These and other demographic characteristics are presented in Table 1.

### 2.2. Materials

The survey questionnaire included 79 items with binary, categorical, and 4- and 5-point Likert scale response types, as well as open-ended questions. A total of 71 items were included for the purpose of the current paper. 

The dependent variables in this study were depression, anxiety, and distress. Depression was measured by the 9-item Patient Health Questionnaire (PHQ-9) instrument [24]. An example would be: “Trouble falling or staying asleep, or sleeping too much”. The respondents were asked to rate themselves on a 4-point Likert scale from 0 (not at all) to 3 (nearly every day). The total score for an individual was obtained by summing up their scores on all items. A score of 15 or above on PHQ-9 indicates moderately severe depression. Cronbach’s alpha for PHQ-9 was obtained and it was 0.88, indicating satisfactory reliability of the scale in the current study.

Anxiety was measured by the 7-item Generalized Anxiety Disorder (GAD-7) instrument [25]. An example would be: “Becoming easily annoyed or irritable”. Respondents were asked to rate themselves on a 4-point Likert scale from 0 (not at all) to 3 (nearly every day). A total score of 15 or above denotes severe anxiety. Cronbach’s alpha for GAD-7 was obtained and it was 0.94, indicating satisfactory reliability of the scale in the current study.

Distress was measured by the 22-item Impact of Event Scale-Revised (IES-R) instrument, which was developed to gauge people’s distress level in response to a specific traumatic event [26], in this case, the COVID-19 pandemic. An example would be: “Any reminders brought back feelings about it”. Respondents rated themselves on a 5-point Likert scale from 0 (not at all) to 4 (extremely). A total score of 33 on IES-R indicates extreme distress. Cronbach’s alpha for IES-R was obtained and it was 0.95, indicating satisfactory reliability of the scale in the current study.

Binary variables for depression, anxiety, and distress were created by using YES for a participant whose PHQ-9 total score was 15 or above, whose GAD-7 score was 15 or above, and whose IES-R score was 33 [24,25,26]. 

A number of independent variables were used in this study. They included eight demographic items, such as age, gender, education, ethnicity, number of children, marital status, occupation, and leadership position. Six other factors included: supervisor communication frequency (daily, 3–4 times a week, 1–2 times a week, biweekly, monthly); type of shift (8 h day shift, 12 h day shift, 12 h night shift); number of missed shifts since the pandemic began (0 shifts, 1–2 shifts, 3–4 shifts, 5–6 shifts, 7 shifts and greater); workplace characteristics (direct exposure to COVID-19 patients, direct exposure to a patient under investigation for COVID-19, direct exposure to the public when entering the hospital through emergency department, indirect exposure to COVID-19 patients, such as equipment contaminated with COVID-19, no exposure to COVID-19 patients); information regarding COVID-19 diagnosis of family members, friends, colleagues, and self; risk of becoming infected with COVID-19 (extremely low, low, moderate, high, and extremely high); and concern of infecting family members when they go home after work (NA—I live alone, extremely low to extremely high). 

Additionally, participants were asked to rate the degree of their concern of becoming infected with COVID-19 on the following items: 1. there is no vaccine for COVID-19 yet, 2. COVID-19 is highly contagious, 3. poor risk control procedures implemented by the hospital for the position, 4. limited availability of PPE, 5. direct contact with COVID-19 patients, and 6. long work hours or working extra shifts. The responses were on a 4-point Likert scale: 0 (strongly insignificant) to 3 (strongly significant). 

Moreover, the survey included two binary questions on work change and one binary question on lifestyle change due to COVID-19: 1. “Has the COVID-19 pandemic changed how you work?”; 2. “Have any of the procedures implemented by the health system due to the COVID-19 pandemic affected how you work?”; 3. “Has your lifestyle changed at all due to COVID-19”? 

Additionally, two open-ended questions asked the participants: “When you go home after work what do you do to prevent potentially exposing your family to COVID-19?” and to “Provide an example of a procedure implemented by the health system due to the COVID-19 pandemic that affected how you work”.

The last part of the survey asked the participants whether they sought and received any psychological resources and services, and if so, what types of psychological services were received. The resources and services included online media, news, or various online media platforms, such as psychological assistance methods and techniques, and psychological resources provided by the health system, such as leaflets, brochures, emails, websites, and books.

### 2.3. Procedure

This study was approved by the local institutional review board. Data were collected from HCWs who worked at the hospital integrated healthcare system located in a southeastern state. The main instrument used to collect data was a cross-sectional, web-based survey via Qualtrics. The online survey was distributed through multiple channels, such as flyers with QR code for immediate access to the survey, emails with links to the survey, and verbal communications. Data collection occurred between 1 June and 15 October 2020, the period corresponding to the days, weeks, and months immediately preceding the first COVID-19 infection peak in southeastern US and therefore associated with the first highest healthcare system utilization level. This period also corresponded with the system providing psychological services to the staff, such as counseling or psychotherapy (including individual or group therapy) and supplying psychological resources (leaflets, brochures, emails, websites, and books). All healthcare workers were eligible to participate in the study. Participation was voluntary, and the responses were anonymous. The online consent was obtained from the participants before they proceeded to the survey questionnaire. 

### 2.4. Data Analysis

Qualtrics data were exported to Minitab version 19 (Windows) (Minitab, LLC, State College, PA, USA) for analysis. Descriptive statistics were calculated first. Chi-Square analysis was conducted to explore the association of the independent variables with the binary dependent variables, including depression concern, anxiety concern, and distress concern. Multivariate binary logistic regression models were created to identify significant factors with good predictive outcomes of the mental health concerns. A stepwise regression method was applied to select the best regression model after examining the association between the independent variables. Additionally, responses to the open-ended questions were item analyzed by counting the frequency of the responses.

## 3. Results

### 3.1. Demographic Characteristics

Overall, 30.1% of participants reported depression symptoms, 28.7% exhibited anxiety symptoms, and 39.4% showed distress symptoms. The prevalence of binary outcomes, namely depression concern, anxiety concern, and distress concern, stratified by gender and age, is shown in Table 2. Among the female participants, 30.2% had depression concern, 29.8% had anxiety concern, and 40.1% showed a distress concern. These percentages are higher in each mental health area compared to the male participants, among which 26.5%, 11.8%, and 35.5% showed depression, anxiety, and distress concern, respectively. The proportion of people who reported symptoms of depression seems to be more prevalent in the younger population (ages 20 to 29) compared to older population group (ages 50 and above). The same pattern applies to the anxious symptomatology. 

### 3.2. COVID-19 Concerns

The COVID-19-related concern scores are presented in Table 3. The top concern was “COVID-19 is highly contagious”, (3.5/4 in degree of concern), while “Poor risk control procedures implemented by the hospital for my position” was the lowest (2.4/4).

### 3.3. Mental Health and Associated Factors (Chi-Square)

We conducted Chi-Square tests (with a significance level α = 0.05) to determine how mental health concerns, symptoms of depression, symptoms of anxiety, and general distress are impacted by different factors, such as demographic characteristics, external interventions, as well as psychological and behavioral changes due to COVID-19. Table 4 contains detailed information on the significant factors based on the *p*-value of the Chi-Square test. Seven factors: age, risk of contracting COVID-19, concern of exposing family to COVID-19, healthcare procedure change, lifestyle change, number of missed shifts since COVID-19, and access to psychological services, were found to be strongly correlated with both depressive and anxious symptomatology. Two additional factors, gender and whether the participants have friends diagnosed with COVID-19, were significantly associated with the anxiety concern. However, no factor was shown to be statistically significant in distress concern based on the current data.

### 3.4. Risk Factors of Mental Health Outcome (Logistic Regression)

Binary logistic regression models were built for predicting the depression concern and anxiety concern separately based on the list of significant factors identified using Chi-Square analysis. After examining the association between the independent variables and selecting a good set of potential factors, the stepwise regression method was applied to choose the best regression model. The common factors in both logistics regression models are psychological services, healthcare procedure change, and concern of exposing family to COVID-19. The odds ratios of each factor in the two binary logistic regression models are presented in Table 5 (for depression) and Table 6 (for anxiety). The area under the receiver operating characteristic (ROC) curve is 0.75 for the depression binary logistic model and 0.73 for the anxiety regression model. 

In the multivariate analysis, we found that HCWs who specified that the procedures implemented by the health system due to COVID-19 affected their work were 7.85 times more likely to report depressive symptoms (95% CI: 2.30–26.72, *p* = 0.001) compared to those who did not. HCWs who received psychological services/resources available through online media, TV news, or various online platforms media; psychological resources (leaflets, brochures, emails, websites, and books) provided by the healthcare system; and counseling or psychotherapy (including individual or group therapy) through the system were 3.58, 2.96, and 1.72 more likely to report depressive symptoms, respectively, compared to those who did not receive any services (95% CI: 1.17–10.96, *p* = 0.025; 95% CI: 1.02–8.53, *p* = 0.045; 95% CI: 0.59–5.03, *p* = 0.323). Additionally, HCWs who had high concern of exposing family to COVID-19 were approximately 3.16 times more likely to report depressive symptoms (95% CI is 1.00–9.92 with *p* = 0.049) compared to those who had extremely low concern. Similarly, HCWs who had extremely high concern of exposing family to COVID-19 were approximately 5.34 times more likely to report depressive symptoms (95% CI is 2.12–13.44 with *p* < 0.001) compared to those who had low concern. 

Similar findings apply to anxiety. HCWs who received psychological services or resources available through online media, TV news, or various online platforms; psychological resources (leaflets, brochures, emails, websites, and books) provided by the healthcare system; and counseling or psychotherapy (including individual or group therapy) through the system were 5.82, 3.81, and 3.83 more likely to report anxiety symptoms, respectively, compared to those who did not receive any services (95% CI: 1.86–18.16, *p* = 0.002; 95% CI: 1.38–10.58, *p* = 0.010; 95% CI: 1.33–11.02, *p* = 0.013). Additionally, HCWs who specified that the procedures implemented by the health system due to COVID-19 affected their work were 2.96 times more likely to report anxiety symptoms (95% CI: 1.20–7.28, *p* = 0.018) compared to those who did not. HCWs who had high concern of exposing family to COVID-19 were approximately 2.25 times more likely to report anxiety symptoms (95% CI is 0.71–7.17 with *p* = 0.046) compared to those who had extremely low concern. Similarly, HCWs who had extremely high concern of exposing family to COVID-19 were approximately 3.67 times more likely to report anxiety symptoms (95% CI is 1.52–8.86 with *p* = 0.004) compared to those who had low concern. 

### 3.5. Qualitative Analysis

The responses from the two open-ended questions “When you go home after work what do you do to prevent potentially exposing your family to COVID-19?” and “Provide an example of a procedure implemented by the health system due to the COVID-19 pandemic that affected how you work” were analyzed. First, the frequency of responses to each question was calculated. The responses were content analyzed first by two members of the research team individually and then collectively discussed before the researchers decided on emerging themes from the responses. 

**Figure 1:**
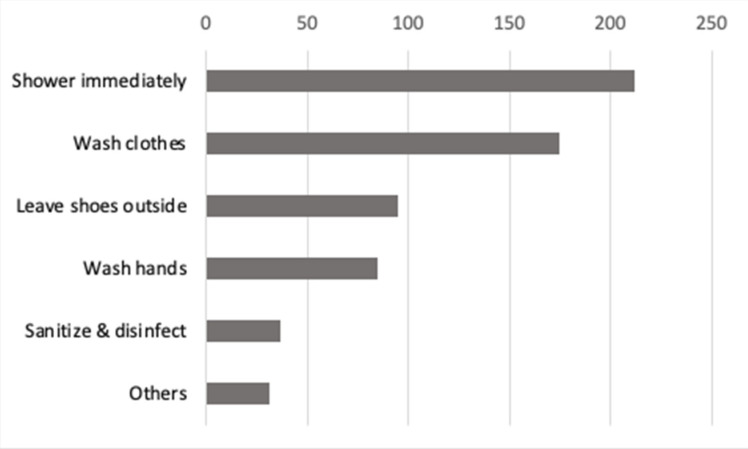
Themes and counts of what HCWs do when returning home from work to prevent exposing family to COVID-19.

The results indicate that most respondents “shower immediately” and “wash clothes” when they go home after work to prevent potentially exposing their family to COVID-19 (Figure 1). To a lesser extent, many indicated that they “Leave shoes outside”, “Wash hands”, and “Sanitize and disinfect”. 

**Figure 2:**
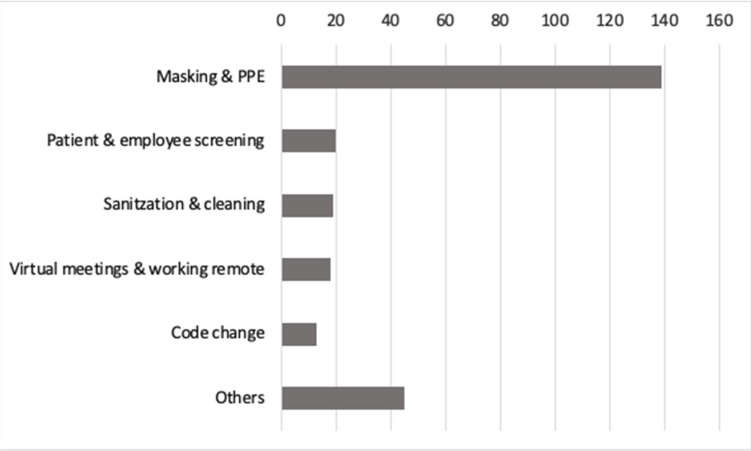
Themes and counts of procedure changes due to COVID-19.

Additionally, the majority of the responses to the open-ended question “Provide an example of a procedure implemented by the health system due to the COVID-19 pandemic that affected how you work” involved the additional precautionary measures put in place by the health system of requiring PPE and masking (Figure 2). Other examples included employee screening, sanitization, working virtually, and code change.

## 4. Discussion

In this study, we investigated the mental health of HCWs and the associated risk factors in a large health system in the suburbs of southeastern US during the first COVID-19 infection peak (1 June 2020 to 15 October 2020), which was associated with the first highest healthcare system utilization level. Additionally, during this period the health system provided psychological services to the staff, such as counseling or psychotherapy (including individual or group therapy) and psychological resources (leaflets, brochures, emails, websites, and books). To the best of the authors’ knowledge, this is one of the first studies that examines the association of nontraditional factors, such as access to psychological services, communication frequency of supervisors, missed shifts, and procedural, work, and lifestyle change due to COVID-19 on the mental health of HCWs during the first peak of the COVID-19 pandemic. 

Specifically, approximately 29% of participants reported anxiety symptoms (GAD-7 ≥ 15), 30% reported symptoms suggestive of moderate or higher depressive symptoms (PHQ-9 ≥ 15), and 39% reported PTSD symptoms (IESR ≥ 33). These results are similar to a previous study conducted in Italy [18] and are less severe than those conducted in China [15,16,20], which mainly involved nurses and doctors. 

In our study, age was the only demographic factor that was significantly associated with both the anxiety and depression symptoms, with younger populations being more prone to anxiety and depression symptoms. Gender was also statistically associated with anxiety, with female HCWs more likely to suffer from anxiety, which is consistent with previous research findings [11,16,17,20]. These gender differences reflect the gender composition of the organization. 

Active and frequent communication is essential in any crisis, especially during a pandemic. We found that communication frequency was not associated with anxious, depressive, or general distress symptoms in our data. This finding could suggest that the health system response moderated the emotional impact of the pandemic on its constituents, perhaps because frequent and active communication was one of the top policies implemented by the health system during the early stages of the pandemic. In addition, senior management provided daily information and updates on COVID-19 through rounding, being visible, sending emails, and offering emotional support (via personal communication). Other studies have found that those who received frequent and trustworthy communication from leaders expressed less anxiety, stress, and burnout [22,27,28].

Working in a health system during the COVID-19 pandemic is inherently stressful [16,20,22]. We found that HCWs’ concerns about personal infectivity were associated with higher levels of both anxious and depressive symptoms. HCWs expressed that their top major concern is “COVID-19 is highly contagious” followed by “Direct contact with COVID-19 patients”, “There is no vaccine for COVID-19 yet”, “Limited availability of PPE”, “Long work hours or working extra shifts”, and “Poor risk control procedures implemented by the hospital for my position”, subsequently. The participants identified other concerns via free text, such as “Co-worker safety compliance”, “Short staffed”, and “Changes in CDC guidelines”. Moreover, many were concerned about exposing family members to COVID-19, and this factor was also an independent predictor for both anxiety and depression symptoms. Participants identified that they “Shower Immediately”, “Wash Clothes”, “Leave Shoes outside of house”, and “Wash Hands” when they go home after work to prevent potentially exposing their families to COVID-19. These results, to some extent, mirror the results found in a study conducted in Poland [21] where “Fear for my health” and “Fear for the loved ones” were statistically associated with mental concerns (GHQ-28) for the medical professionals group, and only “Fear for my health” was statistically associated with mental health concerns (GHQ-28) for the nonmedical medical professionals group. 

Similar to Dohrn et al. [29], this study found that perceived procedural changes implemented by the health system due to COVID-19 was also one of the significant independent factors to predict anxiety and depression. Participants expressed that the procedural changes primarily involved “masking and PPE policies”, “increased sanitization requirements”, “employee screening”, “virtual meetings”, and “code change”. It is possible that the daily struggle to follow constantly changing infection control precautions and the additional steps taken to comply with these rapidly evolving standards were causing HCWs anxiety and depression symptoms. 

Additionally, our findings showed that both the number of missed shifts and lifestyle changes were significantly associated with both anxious and depressive symptoms. One may interpret missed shifts as a first sign of a HCW experiencing mental health disorders or burnout. It has been documented that missed nursing care is associated with burnout and job dissatisfaction among nurses in nursing homes [30]. Alternatively, the number of missed shifts may be attributable to the much stricter quarantine of 14 days for employees in the beginning of the pandemic versus 5 days more recently. 

Equally noteworthy are the factors outside of healthcare altogether that moderated the experience of psychological distress throughout the pandemic. Many HCWs incurred additional psychosocial stressors that contributed to undesirable changes in routine—well-established factors associated with increased distress [31,32]. The various sources of change caused by COVID-19 on HCWs, ranging from how they work and interact with patients and co-workers to lifestyle and social changes, seem to have a negative effect on their mental well-being.

Our results from the bivariate and multivariate analysis showed that there is a significant association between mental health disorders and seeking psychological services, which is expected. It is reasonable to deduce that HCWs with higher acuity of depressive and anxious symptomatology were more likely to seek professional support. It is also possible that people who experience depression and anxiety are more aware of mental health issues and, therefore, tend to seek help. This finding supports previous results by Drew and Matthews [33] that individuals seeking psychological services were more likely to report moderate to severe depression and anxiety. That access to such resources was promoted and made available by the organization might be considered a pragmatic and positive outcome. Future research might investigate the potential effects of such access as it relates to healthcare worker retention, improved occupational functioning, and symptom mitigation in comparison to employees who did not seek professional mental health support. 

Further, it is notable that less traditional methods of accessing resources (e.g., online versus in person) were utilized with higher prevalence. Of concern is that approximately 24% of participants who reported symptoms of anxiety and/or depression did not access the psychological services and resources made available to them by the health system. This suggests perhaps that health systems need to provide additional services, such as on-site and just-in-time (immediate access when needed) counseling. Moreover, health systems have an opportunity to overtly challenge the stigma associated with accessing mental health resources to normalize uptake and infuse self-care into the fabric of the organizational culture. More research is needed to understand the factors that impact healthcare worker utilization of psychological services and to identify best practices for implementing these services to improve the mental well-being of HCWs during a pandemic. 

This study has a few limitations. First, the scope and number of survey participants were relatively limited. The survey was conducted at one healthcare system, which consists of multiple hospitals within a specific state, thus some of our findings may not be generalizable to other hospitals in different regions. Second, we used an online survey to collect data and observed relatively low survey response rate, which may lead to selection bias, as some of the non-participants may have been too stressed to respond or were not interested in participating. Additionally, our study lacks a longitudinal follow-up on the mental health of HCWs. Moreover, it is noteworthy to mention that there are no pre-pandemic baselines for comparison. There are, however, studies that suggest a prevalence of depressive and anxious symptoms among HCWs under normal circumstances ranging between 24 and 26% [34]—findings that are generally comparable to our own analyses. 

## 5. Conclusions

HCWs are pivotal to the community’s successful survival during epidemics and pandemics. In this cross-sectional online survey of HCWs (medical and non-medical) during the first peak of COVID-19, HCWs reported moderate rates of anxiety, depression, and distress symptoms. In addition to the common risk factors that have been previously identified by other researchers, our results suggest the personal risk of contracting COVID-19, HCWs’ concern of exposing family members to COVID-19, number of missed shifts, healthcare procedure changes due to COVID-19, and lifestyle changes due to COVID-19 are all associated with elevated depression and anxiety symptoms among HCWs. Additionally, our findings emphasize the importance of providing additional training and support with PPE and best practices to reduce the spread of COVID-19 to family members after going home from work. Moreover, our findings shed timely light on the importance of providing the proper psychological interventions to promote mental health well-being for HCWs during the stressful COVID-19 pandemic. 

## Figures and Tables

**Table 1:** Demographic characteristics.

Independent Variable	Category	N	%
Gender	Female	348	89.69
	Male	34	8.76
	Missing	6	1.55
Age	20–29	36	9.28
	30–39	82	21.13
	40–49	104	26.80
	50–59	113	29.12
	60+	53	13.66
Number of Children	0	90	23.20
	1–2	189	48.71
	3–4	95	24.48
	4+	13	3.35
Marital Status	Single	135	16.49
	Married/domestic partner	252	64.95
Ethnicity	Caucasian	276	71.13
	African American	77	19.85
	Asian/Pacific Islander	16	4.12
	Others	16	4.12
Education Level	High school or less	25	6.44
	Associate degree	99	25.52
	Bachelor	146	37.63
	MS (equivalent)	89	22.94
	Doctorate	12	3.09
	Others	15	3.87
Occupation	Administration	49	12.63
	Ethicists	25	6.44
	Radiology	33	8.51
	Registered nurse	212	54.64
	Others (Physician, PA, tech, lab, pharmacy, dietician, PT)	68	17.53
Leadership Position	Yes	137	35.31
	No	251	64.69

**Table 2:** Percentage of depression, anxiety, and distress stratified by gender and age.

		Gender		Age				
		Male	Female	20–29	30–39	40–49	50–59	60+
Depression	Yes	26.5%	30.2%	47.2%	31.7%	34.0%	25.9%	17.3%
	No	73.5%	69.8%	52.8%	68.3%	66.0%	74.1%	82.7%
Anxiety	Yes	11.8%	29.8%	44.4%	36.3%	34.3%	20.0%	12.2%
	No	88.2%	70.2%	55.6%	63.8%	65.7%	80.0%	87.8%
Distress	Yes	35.5%	40.1%	31.4%	32.4%	40.0%	49.0%	35.4%
	No	64.5%	59.9%	68.6%	67.6%	60.0%	51.0%	64.6%

**Table 3:** COVID-19 concern scores.

COVID-19 Infection Concerns	Average Degree of Concern (Out of 4)	95% CI Degree of Concern
COVID-19 is highly contagious.	3.5	(3.4, 3.6)
Direct contact with COVID 19 patients.	3.1	(3.0, 3.2)
There is no vaccine for COVID-19 yet.	2.8	(2.7, 2.9)
Limited availability of PPE.	2.8	(2.7, 2.9)
Long work hours or working extra shifts.	2.5	(2.4, 2.6)
Poor risk control procedures implemented by the hospital for my position.	2.4	(2.3, 2.5)

**Table 4:** Chi-Square associate of risk factors (number/percentage %).

Factor	Category	Depression		*p*-Value	Anxiety		*p*-Value
		No	Yes		No	Yes	
Age	20–29	19 (52.78)	17 (47.22)	0.029	20 (55.56)	16 (44.44)	0.001
	30–39	56 (68.29)	26 (31.71)		51 (62.20)	29 (35.37)	
	40–49	64 (61.54)	33 (31.73)		67 (64.42)	35 (33.65)	
	50–59	83 (73.45)	29 (25.66)		88 (77.88)	22 (19.47)	
	60+	43 (81.13)	9 (16.98)		43 (81.13)	6 (11.32)	
Gender	Male	-	-		30 (88.24)	4 (11.76)	0.026
	Female	-	-		238 (68.39)	101 (29.02)	
Risk of contracting COVID-19	Low	65 (84.42)	12 (15.58)	<0.001	64 (83.12)	11 (14.29)	<0.001
	Moderate	118 (69.82)	45 (26.63)		127 (75.15)	39 (23.08)	
	High	82 (57.75)	57 (40.14)		78 (54.93)	58 (40.85)	
Concern of exposing family to COVID-19	N/A (Live alone)	15 (57.69)	10 (38.46)	<0.001	15 (57.69)	10 (38.46)	0.001
	Extremely low	24 (82.76)	5 (17.24)		24 (82.76)	5 (17.24)	
	Low	61 (87.14)	9 (12.86)		57 (81.43)	11 (15.71)	
	Moderate	93 (71.54)	32 (24.62)		96 (73.85)	30 (23.08)	
	High	40 (57.14)	29 (41.43)		43 (61.43)	24 (34.29)	
	Extremely high	32 (50.79)	29 (46.03)		34 (53.97)	28 (44.44)	
Healthcare procedure changes impact work	Yes	185 (67.27)	85 (30.91)	<0.001	188 (68.36)	79 (28.73)	0.018
	No	49 (87.50)	4 (7.14)		48 (85.71)	8 (14.29)	
	Unsure	19 (50.00)	18 (47.37)		23 (60.53)	15 (39.47)	
Lifestyle change due to COVID-19	No	7 (87.50)	1 (12.50)	0.001	8 (100.00)	0 (0.00)	<0.001
	Yes, minimally	44 (84.62)	7 (13.46)		43 (82.69)	7 (13.46)	
	Yes, moderately	82 (73.87)	25 (22.52)		93 (83.78)	16 (14.41)	
	Yes, significantly	117 (60.00)	74 (37.95)		112 (57.44)	79 (40.51)	
Number of Missed Shifts since COVID-19 outbreak	0	199 (75.67)	58 (22.05)	<0.001	196 (74.52)	60 (22.81)	<0.001
	1–2	31 (57.41)	23 (42.59)		36 (66.67)	18 (33.33)	
	3–4	15 (57.69)	10 (38.46)		18 (69.23)	8 (30.77)	
	5+	10 (34.48)	17 (58.62)		10 (34.48)	18 (62.07)	
Access to Psychological Services	Online resources	6 (37.50)	10 (62.50)	<0.001	5 (31.25)	11 (68.75)	<0.001
	Hospital resources	8 (44.44)	10 (55.56)		8 (44.44)	10 (55.56)	
	Hospital psychotherapy	9 (50.00)	8 (44.44)		7 (38.89)	10 (55.56)	
	Did not seek	228 (72.38)	79 (25.08)		237 (75.24)	71 (22.54)	
Friends diagnosed with COVID-19	Yes	-	-		149 (65.35)	73 (32.02)	0.029
	No	-	-		120 (75.00)	35 (21.88)	

**Table 5:** Risk factors for depression by binary logistics regression.

Variable	Value	Reference	OR (95%CI)	*p*-Value	
				Category	Overall
Psychological services	Online resources	Did not seek	3.58 (1.17–10.96)	0.025	0.029
	Hospital resources	Did not seek	2.96 (1.02–8.53)	0.045	
	Psychotherapy	Did not seek	1.72 (0.59–5.03)	0.323	
Healthcare procedure changes impact work	Yes	No	7.85 (2.30–26.72)	0.001	<0.001
	Unsure	No	17.18 (4.25–69.48)	<0.001	
Concern of exposing family to COVID-19	High	Extremely low	3.16 (1.00–9.92)	0.049	0.012
	Extremely high	Extremely low	3.26 (1.03–10.35)	0.045	
	High	Low	5.34 (2.12–13.44)	<0.001	
	Extremely high	Low	5.53 (2.16–14.16)	<0.001	

**Table 6:** Risk factors for anxiety by binary logistic regression.

Variable	Value	Reference	OR (95%CI)	*p*-Value	
				Category	Overall
Psychological services	Online resources	Did not seek	5.82 (1.86–18.16)	0.002	<0.001
	Hospital resources	Did not seek	3.81 (1.38–10.58)	0.010	
	Psychotherapy	Did not seek	3.83 (1.33–11.02)	0.013	
Healthcare procedure changes impact work	Yes	No	2.96 (1.20–7.28)	0.018	0.025
	Unsure	No	4.52 (1.47–12.92)	0.009	
Concern of exposing family to COVID-19	High	Extremely low	2.25 (0.71–7.17)	0.046	0.012
	Extremely high	Extremely low	3.24 (1.02–10.29)	0.169	
	High	Low	2.55 (1.06–6.13)	0.037	
	Extremely high	Low	3.67 (1.52–8.86)	0.004	

## Data Availability

Some or all data and models that support the findings of this study are available from the corresponding author upon reasonable request.
